# Molecular insights into the role of vitamin D in atopic dermatitis: pathogenesis, diagnosis, and emerging therapies

**DOI:** 10.3389/fimmu.2026.1739412

**Published:** 2026-03-30

**Authors:** Pengyue Liu, Lixia Cui, Ge Peng, Xiuping Han

**Affiliations:** 1 Department of Dermatology, Shengjing Hospital of China Medical University, Shenyang, Liaoning, China; 2 Department of Dermatology and Venereology, the Second Affiliated Hospital of Hainan Medical University, Haikou, Hainan, China; 3 Atopy (Allergy) Research Center, Juntendo University Graduate School of Medicine, Tokyo, Japan

**Keywords:** atopic dermatitis, immune modulation, microbiome, skin barrier, vitamin D

## Abstract

Atopic dermatitis (AD) is a chronic inflammatory skin disease characterized by skin barrier dysfunction, immune dysregulation, and microbial imbalance. Increasing evidence suggests that vitamin D, a secosteroid hormone with pleiotropic effects, plays a key role in modulating the pathogenesis of AD. This review provides a comprehensive overview of the molecular mechanisms through which vitamin D influences skin integrity and immune function, focusing on vitamin D receptor-mediated pathways, genetic and epigenetic modifiers, and the interplay with immune cells and microbiota. We also examine the epidemiological correlations between vitamin D deficiency and AD prevalence, diagnostic considerations including biomarkers and serum 25-hydroxyvitamin D evaluation, and therapeutic strategies involving systemic and topical vitamin D interventions. The review further discusses ongoing controversies regarding optimal dosing and evaluates the limitations of current clinical evidence. Finally, emerging research directions, such as vitamin D–microbiota–skin axis and personalized vitamin D therapy, are proposed as promising avenues for advancing AD management.

## 1 Introduction

Atopic dermatitis (AD) is a common chronic inflammatory skin disorder with increasing global prevalence, particularly among children and adolescents (1). AD is characterized by pruritic eczematous lesions, recurrent flares, and a complex pathophysiology involving epidermal barrier dysfunction, type 2-dominant immune responses, and microbial dysbiosis (2). In recent years, vitamin D has emerged as a critical immunomodulatory and skin-protective factor, with broad implications beyond calcium and phosphate homeostasis. Circulating vitamin D exists predominantly as 25-hydroxyvitamin D [25(OH)D], which includes two main forms: 25(OH)D_2_ (ergocalciferol-derived) and 25(OH)D_3_ (cholecalciferol-derived). Both forms are produced through hepatic hydroxylation of vitamin D_2_ or D_3_ and serve as the major biomarkers for assessing vitamin D status (3). Among them, 25(OH)D_3_, originating mainly from cutaneous synthesis under ultraviolet B (UVB) exposure, is considered the more potent and physiologically relevant metabolite for immune and skin regulation (3). Vitamin D exerts its biological effects primarily through conversion of 25(OH)D to its active form, 1,25-dihydroxyvitamin D [1,25(OH)_2_D], which binds to and activates the vitamin D receptor (VDR). The VDR is widely expressed in keratinocytes, dendritic cells, T cells and other skin-resident immune cells (3). Through this pathway, vitamin D modulates keratinocyte differentiation, tight junction integrity, antimicrobial peptide (AMP) expression and Th1/Th2/Th17 immune balance—all processes intimately involved in AD pathogenesis.

Accumulating evidence points to a potential link between vitamin D deficiency and increased AD severity, particularly in pediatric populations and during winter months when UVB exposure is limited. However, the precise mechanisms through which vitamin D modulates AD pathogenesis remain under active investigation. Furthermore, clinical trials exploring vitamin D supplementation in AD have produced mixed results, likely reflecting differences in baseline deficiency, genetic polymorphisms affecting vitamin D metabolism and disease heterogeneity. This review aims to synthesize current molecular, immunological and clinical evidence on the role of vitamin D in AD, providing a foundation for future therapeutic innovation and precision medicine approaches.

## 2 Epidemiology and pathophysiology of AD and vitamin D deficiency

### 2.1 Pathophysiological mechanisms of AD

The pathophysiology of AD is complex, involving a combination of genetic, immunological and environmental factors. It is characterized by a disrupted skin barrier function and an abnormal immune response. One of the key features of AD is the impairment of the epidermal barrier. Genes related to epidermal differentiation and barrier function, such as filaggrin, are often mutated or dysregulated in AD patients. Filaggrin is essential for maintaining the integrity of the skin barrier, and its deficiency can lead to increased transepidermal water loss, making the skin more susceptible to environmental allergens and irritants (4).

The immune response in AD is also dysregulated. There is an imbalance between T helper (Th) 1 and Th2 cell responses, with a predominance of Th2-type cytokines such as interleukin-4 (IL-4), IL-5, and IL-13. These cytokines promote the production of IgE, eosinophilia and mast cell activation, contributing to the allergic inflammation seen in AD (2). Additionally, Th17 and Th22 cells have also been implicated in the pathogenesis of AD, secreting cytokines like IL-17 and IL-22, which can further exacerbate skin inflammation and impair the skin barrier (5).

Neuroimmune interactions also play a role in AD pathophysiology. Neuropeptides released from sensory nerves can modulate the immune response and skin barrier function. For example, substance P can stimulate the release of cytokines and chemokines from keratinocytes and immune cells, promoting inflammation (6). Moreover, the skin microbiome in AD patients is often dysbiotic, with an over-representation of *Staphylococcus aureus*, which can produce toxins and superantigens that trigger and exacerbate the immune response (7). All these factors interact in a complex manner to drive the chronic inflammation and skin manifestations characteristic of AD.

Importantly, recent advances have highlighted that these pathological processes occur within a broader skin neuro–immuno–endocrine network. The skin is increasingly recognized as an active neuroendocrine organ capable of sensing environmental stimuli, including ultraviolet radiation, temperature, microbial signals and psychological stress, and translating them into coordinated immune and barrier responses. Local production of neuropeptides, cytokines, corticosteroids and secosteroids forms an integrated regulatory system that modulates cutaneous homeostasis (8). Within this framework, vitamin D, synthesized in keratinocytes upon UVB exposure and acting through the VDR, can be considered part of the skin’s intrinsic neuro–immuno–endocrine machinery (8). By influencing keratinocyte differentiation, immune polarization, AMP expression, and inflammatory signaling pathways such as NF-κB signaling, vitamin D may serve as a key mediator linking environmental factors to immune and barrier dysfunction in AD.

### 2.2 Vitamin D deficiency trends in AD

Concurrently, deficiency in circulating 25(OH)D has emerged as a significant public health issue worldwide. Understanding the trends of AD prevalence and low serum 25(OH)D levels is crucial for exploring their potential relationship. The global prevalence of vitamin D deficiency, defined by reduced circulating 25(OH)D concentrations, varies across different populations. A meta-analysis indicated that the prevalence of vitamin D deficiency, defined as serum 25(OH)D levels below 50 nmol, was found to be 59.7% (95% CI 45.9 - 72.1) among the elderly (9). In the context of AD, some studies have suggested that lower serum 25(OH)D concentrations were associated with greater AD severity (10). For instance, a cross-sectional study in a Bangladeshi population living in East London found that 84.3% of the participants with available 25(OH)D_3_ results had deficient or insufficient lowest 25(OH)D_3_ levels, and there was an inverse correlation between lowest 25(OH)D_3_ and Eczema Area and Severity Index (EASI) score (11).

Multiple factors contribute to the high prevalence of low circulating 25(OH)D. Many countries have a relatively low supply of foods rich in vitamin D, and inadequate exposure to natural UVB radiation from sunlight is also a major factor (12). Beyond its classical role in initiating vitamin D synthesis from 7-dehydrocholesterol in the epidermis, UVB acts as an environmental regulator of cutaneous and systemic homeostasis. UVB exposure activates local neuro–immuno–endocrine pathways, inducing the production of proopiomelanocortin-derived peptides, cytokines, corticosteroids and other bioactive mediators that modulate immune balance and barrier integrity (8, 13). These signals may influence both local skin responses and central regulatory axes. Certain populations, such as the elderly, infants, children, adolescents, pregnant or lactating women, may be particularly vulnerable to low circulating 25(OH)D due to reduced synthesis capacity, limited sunlight exposure or altered UVB responsiveness (14). In patients with AD, where barrier dysfunction and neuroimmune dysregulation are central features, reduced UVB exposure may therefore contribute to disease susceptibility and severity through combined effects on systemic 25(OH)D status and broader neuro–immune–endocrine signaling. It should be noted that most epidemiological studies assess circulating 25(OH)D as a biomarker of vitamin D status rather than the biologically active metabolite 1,25(OH)_2_D. Although the causal relationship between low serum 25(OH)D and AD severity remains under investigation, the high prevalence of deficiency and its potential link to AD highlight the need for further well-designed studies.

## 3 Molecular mechanisms of vitamin D in AD

### 3.1 Classical and non-canonical vitamin D signaling in epidermal homeostasis and immune modulation

The biologically active metabolite 1,25(OH)_2_D_3_ plays a pivotal role is crucial in maintaining epidermal homeostasis and modulating immune response, processes that are disrupted in AD. In the classical pathway, activation of the VDR by its ligand 1,25(OH)_2_D_3_ forms a heterodimer with the retinoid X receptor (RXR). The VDR–RXR complex then binds to vitamin D response elements(VDRE) in target genes, regulating their transcriptional programs involved in keratinocyte proliferation, differentiation, and barrier function (15). For example, genes involved in the synthesis of filaggrin, a key protein for skin barrier function, may be regulated by VDR signaling. Given that filaggrin deficiency is common in AD, restoration of proper VDR signaling may contribute to enhance filaggrin expression and improve the skin barrier (16).

Beyond epidermal differentiation, VDR signaling exerts important immunomodulatory effects. VDR is expressed in various immune cells, including macrophages, dendritic cells (DCs) and T cells. In macrophages, treatment with 1,25(OH)_2_D_3_ stimulates the production of AMPs, which is important for defense against skin infections often associated with AD (17). In DCs, 1,25(OH)_2_D_3_ suppresses the production of pro-inflammatory cytokines and promotes the development of tolerogenic phenotype, which are important for maintaining immune tolerance (18, 19). In T cells, exposure to 1,25(OH)_2_D_3_ can lead to the suppression of Th1 and Th17 cell-mediated inflammation and the induction of regulatory T (Treg) cells, thereby modulating the overall immune response in the skin (20).

**Figure 1:**
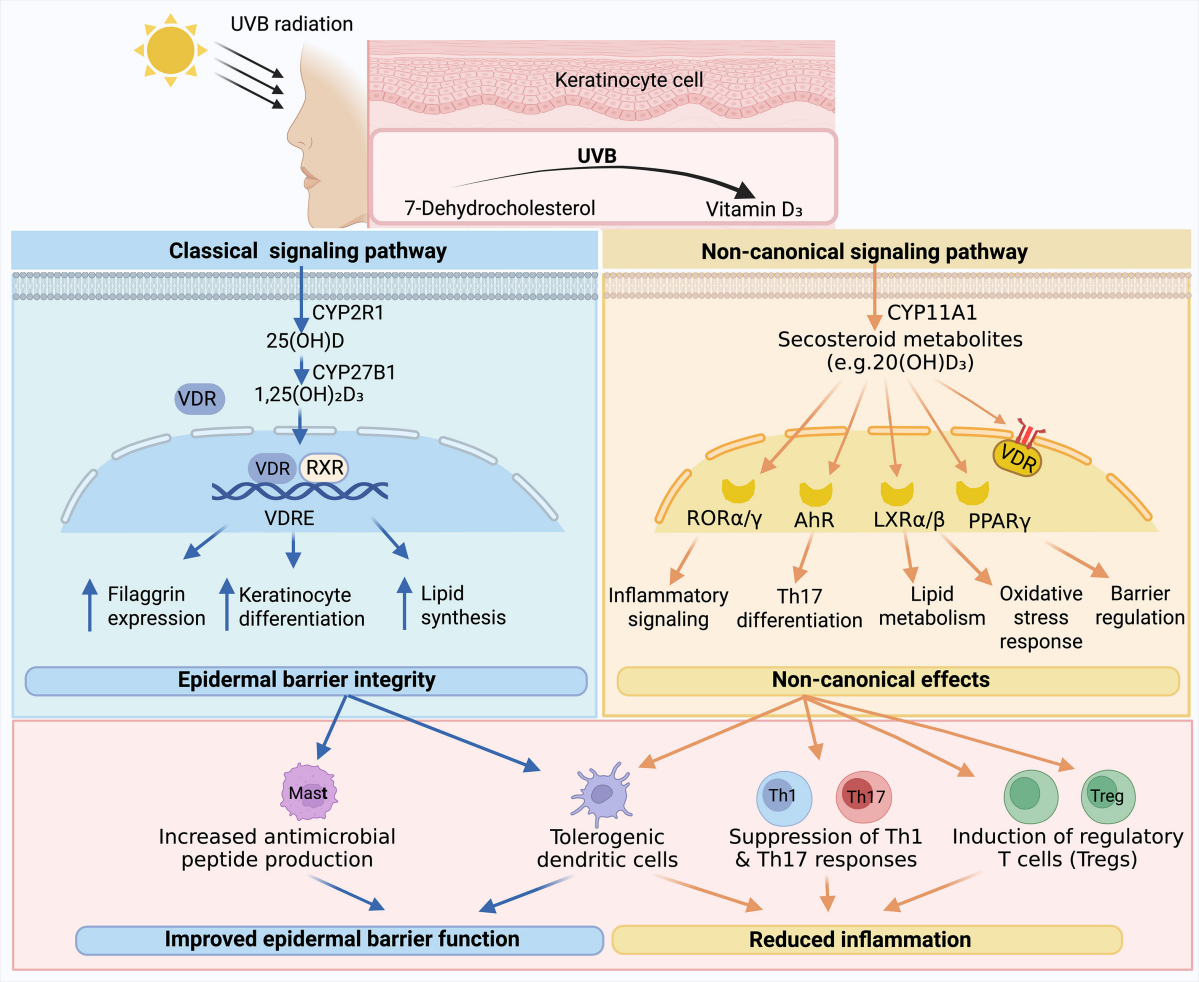
Classical and non-canonical vitamin D signaling pathways in epidermal homeostasis and immune modulation. UVB-induced synthesis of vitamin D_3_ in keratinocytes initiates both classical and alternative activation pathways. The classical pathway involves conversion to 1,25(OH)_2_D_3_, which binds VDR–RXR complexes to regulate genes involved in barrier function and immune modulation. The non-canonical CYP11A1 pathway generates secosteroid metabolites that act via VDR or alternative nuclear receptors such as RORα/γ, AhR, LXR, and PPARγ. These pathways collectively influence keratinocyte differentiation, antimicrobial peptide production, dendritic cell tolerance, and T-cell polarization, contributing to the regulation of AD pathogenesis.

However, emerging evidence indicates that vitamin D biology in the skin extends beyond classical 1,25(OH)_2_D_3_–VDR signaling. The skin expresses CYP11A1, which can generate multiple biologically active secosteroid metabolites, including 20-hydroxyvitamin D_3_(20(OH)D_3_) and related hydroxyderivatives (21). These non-canonical metabolites may function as biased VDR agonists or interact with alternative nuclear receptors, such as retinoic acid-related orphan receptors (RORα/γ), aryl hydrocarbon receptor (AhR), liver X receptors (LXRα/β) and peroxisome proliferator-activated receptor-γ (PPARγ) (21). Some derivatives may also signal via non-genomic VDR sites or membrane-associated receptors. These expanded signaling pathways are particularly relevant to AD. RORγ is a key regulator of Th17 differentiation (22), AhR influences barrier immunity and microbial responses (23, 24), and PPARγ modulates lipid metabolism and epidermal differentiation (25, 26). Thus, CYP11A1-derived secosteroids may regulate inflammatory and barrier pathways in AD through mechanisms that are partially independent of classical VDR genomic signaling. Incorporating the classical and non-canonical pathways broadens the mechanistic framework of vitamin D action in AD and highlights potential novel therapeutic targets (**Figure 1**).

### 3.2 Genetic and epigenetic modifiers of vitamin D activity in AD

Genetic polymorphisms in genes related to vitamin D metabolism and action have been associated with AD. For example, a study found that the *Cyp24a1* rs2248359-major C allele was significantly over-represented in patients with AD compared with controls, especially in those with severe AD (27). The *Cyp24a1* gene encodes an enzyme involved in vitamin D inactivation, and this polymorphism may affect the metabolism and activity of vitamin D in AD patients. Another study investigated four common *VDR* gene polymorphisms in AD patients and found that in patients with severe AD, the *VDR BsmI* (rs1544410) G allele, *ApaI* (rs7975232) C allele, and *TaqI* (rs731236) T alleles were over-represented compared with healthy controls (28). These alleles may influence the function of VDR and thus the response to vitamin D in AD.

Epigenetic modifications also play a role. Prenatal 25(OH)D deficiency has been shown to affect the development of AD via DNA methylation (29). DNA methylation can alter the expression of genes related to AD pathogenesis, potentially through its impact on vitamin D-related pathways (29). Additionally, studies have explored the relationship between plasma levels of bioactive vitamin D and its ligands and AD severity. Plasma 25(OH)D_3_ levels showed a negative correlation with eosinophils in blood and SCORing Atopic Dermatitis (SCORAD) values in AD patients, while 1,25(OH)_2_D_3_ and 9-cis-13,14-dihydroretinoic acid levels correlated positively with plasma IgE, eosinophils and SCORAD values, suggesting that the metabolic activation of vitamin D and co-liganding of RXR may be important signaling mechanisms in AD (30).

### 3.3 Vitamin D and its crosstalk with immune cells in AD

The active metabolite 1,25(OH)_2_D_3_ interacts with various immune cells in the context of AD, influencing the disease’s immunopathogenesis. DCs are key players in the initiation of the immune response, and vitamin D signaling can modulate their function. In children with AD, lower circulating 25(OH)D levels has been associated with an increased allergic phenotype of circulating DCs. Ex vivo, 1,25(OH)_2_D_3_ significantly decreased the expression of FcϵRI on myeloid DCs (mDCs) and surface-bound IgE on mDCs and plasmacytoid DCs (pDCs). Oral vitamin D supplementation also reduced the expression of surface-bound IgE on pDCs in these children, suggesting a potential mechanism by which vitamin D supplementation may improve AD severity (31).

T cells are another important target of vitamin D–mediated regulation. In a clinical study of children with AD, vitamin D supplementation normalized the Th1 and Th2 cytokine profiles. The initial levels of cytokines such as IL-2, IL-4, IL-6 and IFN-γ were higher in AD patients compared to healthy controls, and after 3 months of vitamin D supplementation, there was a significant reduction in these cytokines along with a decrease in the AD severity as measured by the SCORAD index (32). Moreover, topical application of 1,25(OH)_2_D_3_ has been shown to increase the proportion of Treg cells in the skin of mice with contact dermatitis, a model related to AD (33). This indicates that vitamin D can modulate the T-cell-mediated immune response in AD. Moreover, IL-36G-expressing monocytes play a crucial role in AD (34). Meanwhile, 1,25(OH)_2_D_3_ inhibits monocyte/macrophage proinflammatory cytokine production by targeting MAPK phosphatase-1 (35). Although the direct impact of vitamin D metabolites on IL-36G-expressing monocytes in AD is yet to be fully elucidated, given vitamin D’s general immunomodulatory effects, it may potentially influence the activation and cytokine production of IL-36G-expressing monocytes in AD. Overall, these findings indicate that systemic vitamin D status—reflected by serum 25(OH)D levels—may influence local immune regulation through conversion to 1,25(OH)_2_D_3_ in immune cells, thereby shaping DC function, T-cell polarization, and monocyte-mediated inflammation in AD.

### 3.4 The role of vitamin D in skin barrier in AD

In terms of skin barrier function, vitamin D has been shown to influence the differentiation and proliferation of keratinocytes (36). In a study on mice, calcifediol, the active metabolite of vitamin D, was found to reduce spinous layer thickening, downregulate aquaporin 3 (AQP3) expression, and restore the barrier function of the skin in a 2,4-dinitrochlorobenzene (DNCB)-induced AD mouse model (37). Vitamin D may also enhance the expression of tight junction proteins in the skin, which are important for maintaining the integrity of the skin barrier (38). Additionally, vitamin D stimulates the expression of AMPs such as cathelicidin in macrophages and dendritic cells (18). Notably, cathelicidin has antibacterial, antiviral, skin barrier enhancing and immunomodulatory properties, which can help combat skin infections and improve skin barrier dysfunction in AD (39, 40). Therefore, the effects of vitamin D on the skin barrier and immune regulation suggest its potential as a therapeutic agent in AD management.

### 3.5 Microbiota–vitamin D–skin axis

The microbiota-vitamin D-skin axis represents an emerging area of research in AD, highlighting the complex interactions between the skin microbiota, vitamin D, and the skin’s immune and barrier functions. Exposure to UVB light, which is important for vitamin D synthesis in the skin, can modulate the human intestinal microbiome. A clinical pilot study showed that in healthy human females, exposure to narrow-band UVB (NB-UVB) light increased serum 25(OH)D levels and significantly increased the alpha and beta diversity of the intestinal microbiota in the group that did not take vitamin D supplements prior to the study (41). This suggests that environmental UVB exposure and skin-derived vitamin D signaling may indirectly shape gut microbial communities.

In the context of AD, dysbiosis of the gut microbiota has been associated with AD (42), and vitamin D may play a role in restoring this balance. Vitamin D can influence the intestinal epithelium and mucosal immune cells, which in turn can shape the microbial communities in the gut. For example, vitamin D can regulate the expression of tight junction proteins on intestinal epithelial cells, which is critical for barrier function in the gut (43). Additionally, vitamin D can support the function of innate lymphoid cells that produce IL-22, suppress IFN-γ and IL-17 by T cells, and induce regulatory T cells in the mucosal tissues, all of which can contribute to maintaining gut homeostasis and potentially impact the skin’s condition in AD (44).

Conversely, the skin microbiota can also interact with vitamin D and the skin’s immune system. In AD, the over-growth of *S. aureus* on the skin can trigger an immune response. Vitamin D may enhance the skin’s innate immune defense against such pathogens through the induction of AMPs including cathelicidin and human defensins (45). These AMPs not only inhibit *S. aureus* growth but also help maintain microbial balance by supporting commensal populations such as *Staphylococcus epidermidis*. Moreover, VDR activation influences epidermal lipid synthesis and barrier repair, creating a microenvironment less permissive to *S. aureus* colonization. Commensal microbes such as *S. epidermidis* can further stimulate AMP production and enhance barrier function, forming a reciprocal host–microbe regulatory loop. Impaired vitamin D signaling may therefore weaken AMP induction, disrupt microbial equilibrium, and facilitate pathogenic dominance in AD.

Taken together, vitamin D appears to modulate both systemic gut-derived immune signals and local cutaneous microbial ecology. Through VDR-dependent regulation of AMPs, barrier integrity, and immune polarization, vitamin D signaling may influence the competitive balance between pathogenic organisms such as *S. aureus* and protective commensals.

## 4 Diagnostic considerations for vitamin D and AD

### 4.1 Clinical assessment of vitamin D status in patients with AD

Assessing the vitamin D status in patients with AD is crucial for understanding the disease’s pathophysiology and potential treatment strategies. Serum 25(OH)D is the most used biomarker to evaluate vitamin D status. Multiple studies have investigated the relationship between 25(OH)D levels and AD severity. A meta-analysis found that the serum 25(OH)D level was lower in AD patients of all ages compared with healthy controls, with a standardized mean difference of −14 nmol/L (95% CI −25 to −2), and this difference was more prominent in pediatric AD patients [standardized mean difference = −16 nmol/L (95% CI −31 to −1)] (46). Another study in Mongolian children with winter-related AD showed that vitamin D supplementation improved the disease severity, as measured by the EASI score. The treated group had a significant increase in 25(OH)D levels, and there was a clinically and statistically significant improvement in the EASI score compared to the placebo group (47). In addition to measuring 25(OH)D levels, other factors may also be considered in the clinical assessment. For example, some studies have explored the relationship between vitamin D levels and the frequency of bacterial skin infections in AD patients, showing that the frequency of bacterial skin infections was higher in AD patients with lower 25(OH)D levels (48). It is important to note that different clinical trials have used distinct severity metrics, including SCORAD, EASI, and Investigator’s Global Assessment. EASI primarily assesses objective inflammatory signs, whereas SCORAD incorporates both objective signs and subjective symptoms such as pruritus. Some studies suggest that vitamin D supplementation may more consistently improve objective signs than subjective symptom scores; however, the available data remain heterogeneous and limited. Overall, clinical assessment of vitamin D status in AD patients can provide valuable information for understanding the disease and guiding potential vitamin D-based therapies.

### 4.2 Biomarkers and endophenotypes in vitamin D and AD

Biomarkers and endophenotypes can offer insights into the complex relationship between vitamin D and AD, helping to better understand the disease mechanisms and potentially predict treatment responses. Cytokine levels are important biomarkers in this context. In children with AD, the levels of cytokines such as IL-2, IL-4, IL-6 and IFN-γ were found to be higher compared to healthy controls. After vitamin D supplementation, there was a significant reduction in these cytokine levels along with a decrease in AD severity as measured by the SCORAD index (49). This suggests that these cytokines may be involved in the immunomodulatory effects of vitamin D in AD.

**Figure 2:**
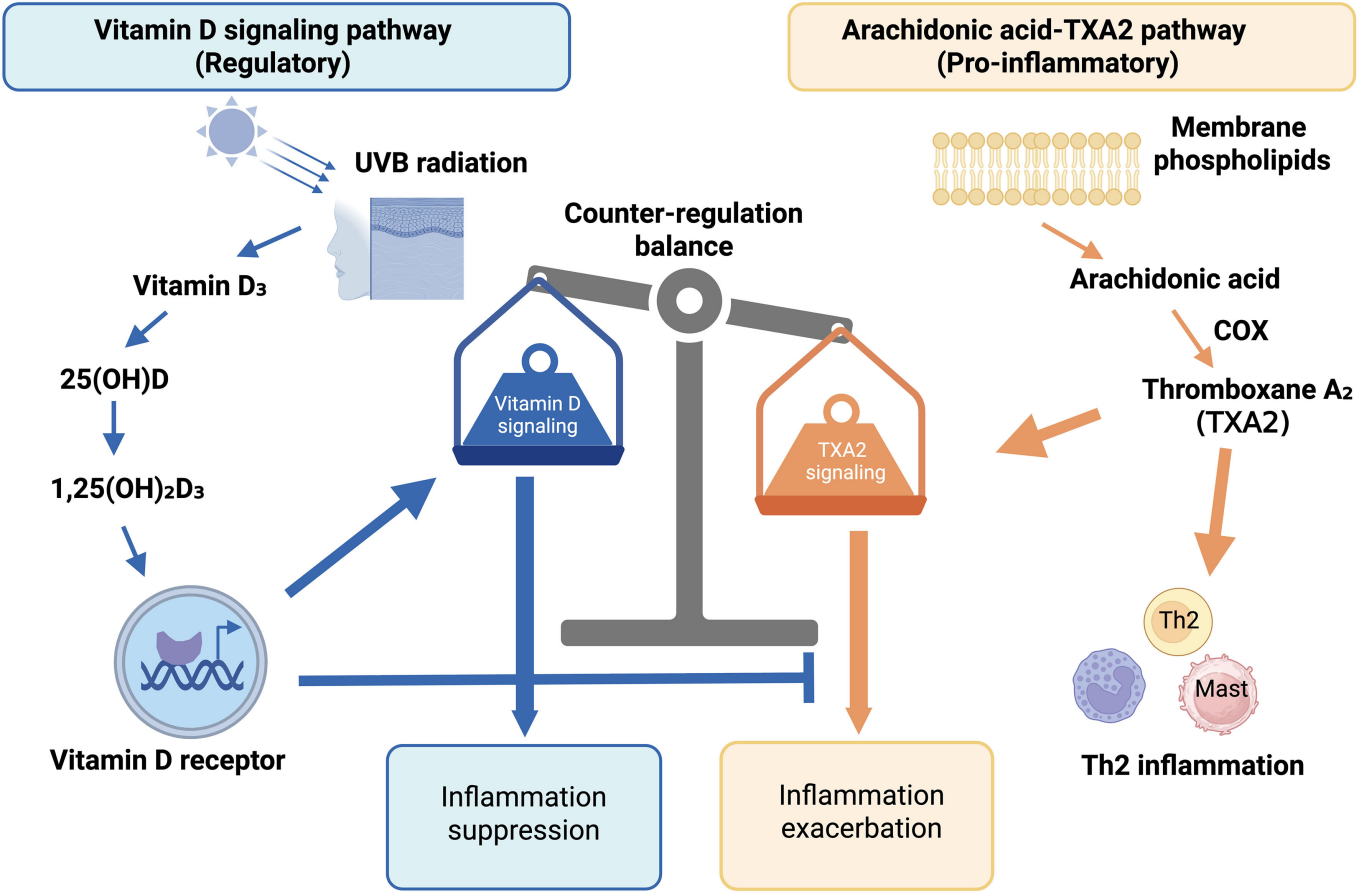
Intersection of vitamin D signaling and the arachidonic acid–thromboxane pathway in AD. This schematic illustrates the conceptual interaction between vitamin D signaling and the arachidonic acid–thromboxane pathway in the pathogenesis of AD. Arachidonic acid is metabolized via cyclooxygenase (COX) to generate thromboxane A2 (TXA2), a lipid mediator that promotes vasoconstriction, endothelial activation, and Th2-skewed inflammation. Elevated TXA2 levels in early life have been associated with increased AD risk and enhanced type 2 immune responses. In parallel, ultraviolet B (UVB)–induced vitamin D synthesis leads to formation of 1,25(OH)_2_D, which activates the vitamin D receptor (VDR). VDR signaling may suppress excessive TXA2 production or related inflammatory amplification pathways. Thus, adequate vitamin D signaling may counterbalance eicosanoid-driven inflammation, whereas vitamin D deficiency may permit enhanced TXA2-mediated immune activation. The diagram highlights a regulatory equilibrium between lipid mediator–driven inflammation and vitamin D–dependent immune modulation in AD.

Eicosanoids, including thromboxanes, prostaglandins, and leukotrienes, are bioactive lipid mediators derived from the arachidonic acid cascade and play important roles in inflammation. In early life, elevated thromboxane A2 (TXA2) levels have been associated with increased risk of developing AD and Th2 inflammation (50). Notably, vitamin D deficiency has been linked to increased TXA2 production and vasoconstriction, while vitamin D supplementation can potentially reduce TXA2 levels and attenuate inflammatory signaling (51). Mechanistically, vitamin D signaling may intersect with the arachidonic acid pathway by modulating cyclooxygenase activity, oxidative stress, and downstream inflammatory mediators (**Figure 2**). This indicates that perturbations in eicosanoid metabolism may be related to the development of AD and could potentially be influenced by vitamin D.

Endophenotypes such as allergic sensitization may also interact with vitamin D in AD. A study found that in children with AD, vitamin D was more strongly correlated with disease severity in those with allergic sensitization. There was a negative correlation between the SCORAD score and serum vitamin D level in the group with allergic sensitization, while no such correlation was seen in the non-sensitized group (52). Understanding these biomarkers and endophenotypes can help in stratifying AD patients and tailoring vitamin D-based treatment strategies.

## 5 Therapeutic strategies targeting vitamin D pathways in AD

### 5.1 Vitamin D supplementation and its efficacy in AD treatment

Vitamin D supplementation has been investigated as a potential treatment for AD, with varying results reported in different studies. Emerging evidence suggests that clinical efficacy may be influenced by dose, baseline vitamin D status, geographic location, and treatment duration. For clarity, available trials can be broadly categorized into low-dose (≤1,000 IU/day), moderate-dose (1,001–2,000 IU/day) and high-dose (>2,000 IU/day) regimens (**Table 1**). Low-dose supplementation (≤1,000 IU/day) has demonstrated variable outcomes. For example, a randomized, double-blind, placebo-controlled trial in children with AD found that after 3 months of vitamin D oral supplementation at a dose of 1,000 IU/day, there was a significant increase in vitamin D levels. Concomitantly, there was a significant reduction in the SCORAD index, as well as in the levels of cytokines such as IL-2, IL-4, IL-6 and IFN-γ, indicating an improvement in AD through the normalization of the Th1 and Th2 inflammatory pattern (53). Another study in Mongolian children with winter-related AD demonstrated that vitamin D supplementation at 1000 IU/day for 1 month led to a clinically and statistically significant improvement in the EASI score compared to placebo (47). However, other low-dose studies have failed to show consistent clinical benefit (57, 58). Moderate-dose regimens (1,600–2,000 IU/day) have yielded somewhat more consistent reductions in AD severity in certain populations, although results remain mixed. A systematic review and meta-analysis including 5 randomized controlled trials (RCTs) found that vitamin D supplementation did not decrease AD severity in general, even when AD was classified as severe vs non-severe (46). However, it was effective in RCTs that included both children and adults, but not in those that included only children (66). High-dose supplementation (>2,000 IU/day, including 4,000–5,000 IU/day regimens) has not uniformly demonstrated superior clinical efficacy compared to lower doses. While some analyses suggest that doses exceeding 2,000 IU/day may be associated with greater reductions in AD severity, other trials have reported minimal or no additional benefit, indicating that dose escalation alone does not guarantee improved outcomes (66). Taken together, current evidence suggests the possibility of a therapeutic threshold near 2,000 IU/day in selected populations, particularly in individuals with baseline deficiency or limited UVB exposure. However, responses appear to be influenced by baseline serum 25(OH)D levels, geographic latitude, seasonal variation, age group and disease severity. Therefore, while vitamin D supplementation remains biologically plausible and generally safe, further dose-stratified and baseline-adjusted randomized trials are required to define an evidence-based therapeutic window for AD.

**Table 1:** Vitamin D therapies in AD clinical trails.

Low-dose supplementation (≤1,000 IU/day) *					
Objectives	% of male/mean age	Intervention/vitamin D dose/formulation	Outcomes	Adverse events	Refs
Children with AD	Not specified/4 years	1,000 IU/day oral cholecalciferol for 3 months	Significant reduction in SCORAD and cytokines (IL-4, IL-6, IFN-γ)	No significant adverse effects	(53)
Mongolian children with AD	59%/9 years	1,000 IU/day oral cholecalciferol for 1 month	Significant EASI score improvement during winter season	Well tolerated	(47)
Patients with AD	Group P (Placebo): 9.1%/26.1 years Group E (Vitamin E): 27.3%/29.0 years	Group E: 600 IU/day oral synthetic all-rac-α-tocopherol (vitamin E) + vitamin D placebo for 60 days	1. SCORAD scores were significantly reduced in Groups E compared to baseline. 2. Significant reduction in objective SCORAD, lichenification, and pruritus.	No significant adverse effects were reported	(54)
Pregnant women and their offsprings	0/31 years	Oral cholecalciferol 1000 IU per day, taken from around 14 weeks’ gestation until deliver	Lower odds ratio of AD at age 12 months; Lower odds ratio of AD weakened and was not statistically significant at ages 24 months or 48 months	Not mentioned	(55)
Pregnant women and their offsprings	0/31 years	Oral cholecalciferol 1000 IU per day, taken from around 14 weeks’ gestation until deliver	Lower odds ratio of AD at age 12 months; Lower odds ratio of AD weakened and was not statistically significant at ages 24 months or 48 months	Not mentioned	(55)
Infants with AD	59.26%/4.87 months	Oral vitamin D3–1400 IU daily for 2 months	The mean SCORAD score was decreased compared to control group	No undesired side effects	(56)
Children with mild AD	55%/median age: 7 years	Ergocalciferol (vitamin D2) 1000 IU once daily for 1 month	No significant group differences were noted	No adverse events	(57)
Mothers of infants with facial eczema at 1 month	0/31.4 years	800 IU oral vitamin D3 per day for 6 weeks	No significant effects on infantile eczema at the 3-month check-up; Contribute to a higher risk for food allergy up to 2 years of age	There were no reports of serious adverse events or hospital admissions in either participating mothers or their infants while mothers were taking the supplement	(58)
Moderate-dose supplementation (1,001–2,000 IU/day)					
Patienst with severe AD	51.2%/11–12 years	1,600 IU/day oral vitamin D3 and topical steroid for 3 months	Greater EASI improvement and higher serum 25(OH)D vs placebo	No major side effects reported	(59)
Patients with AD	Group P (Placebo): 9.1%/26.1 years Group D (Vitamin D): 25%/21.2 years Group DE (Vitamins D & E):27.3%/27.5 years	Group D: 1600 IU/day oral cholecalciferol (vitamin D3) + vitamin E placebo for 60 days Group DE: 1600 IU/day cholecalciferol (vitamin D3) + 600 IU/day vitamin E for 60 days	1. SCORAD scores were significantly reduced in Groups D and DE compared to baseline. 2. Significant reduction in objective SCORAD, lichenification, and pruritus.	No significant adverse effects were reported	(54)
Patients with AD	Not specified/Not specified	Group D: 1600 IU/day oral cholecalciferol (vitamin D3) for 60 days	Significantly improve AD severity	No adverse effects were reported	(60)
Patients with AD	Phase 1: 54.5%/7.4 years Phase 2: 47.6%/8.1 years	Phase 1: Analyze the relationship between serum vitamin D levels and AD severity Phase 2: Oral vitamin D 2000 IU daily for 3 months	Phase 1: patients with lower VD levels had significantly more severe AD Phase 2: unable to significantly improve AD severity	No adverse effects observed	(61)
High-dose supplementation (>2,000 IU/day)					
Patients with moderate-to-severe AD	33%/12.6 years	Oral cholecalciferol (vitamin D3) 5000 IU daily for 12 weeks	80% of patients with serum levels of vitamin D < 20 ng/mL still had moderate-to-severe AD, despite treatment	No cases of hypercalcemia or other safety concerns were reported	(62)
Adult with moderate and severe AD	46.7%/31.2 years	4,000 IU cholecalciferol (vitamin D3) per day for 21 days	No significant change in skin cathelicidin, HBD-3, IL-13, or EASI scores	Five adverse events were reported during this study; none of which were serious or determined to be related to the therapy.	(63)
Adult with moderate and severe AD	Not mentioned	Oral cholecalciferol (vitamin D3) at 4000 IU per day for 21 days	AD lesional skin showed a statistically significant increase in cathelicidin expression; Rajka-Langeland score not mentioned	No subject complained of any adverse event during supplementation	(64)
Patients with moderate–severe AD	33%/12.6 years	5000 IU/day of oral vitamin D3 per day for 12 weeks	Serum levels of 25(OH)D ≥20 ng/ml, regardless of whether or not they had received supplementation, showed a lower SCORAD compared to those with levels <20 ng/ml (deficiency);The majority of patients with a deficiency were associated with higher SCORAD rates or severe AD;Those with deficiency experienced a smaller reduction in their SCORAD compared to the clinical improvement observed in the patients that reached levels ≥20 ng/ml. There were no significant differences between the people with levels ≥20 and ≥30 ng/m	Proved to be safe	(62)
AD diagnosed by Hanifin and Rajka criteria and SCORAD≥10	53%/6.3 years	Weekly oral cholecalciferol (vitamin D3) or placebo for 6 weeks (8000 IU for ages 2–5.9 years; 12,000 IU for ages 6–11.9 years and 16,000 IU for ages 12–17.9 years)	The improvement in SCORAD scores was not statistically significant. No significant differences between groups in the change of eosinophil count, total IgE, specific IgEs against SEA and SEB, or chemokines CCL17, CCL22, CCL27 and LL37; Vitamin D supplementation did not significantly affect rates of *S. aureus* colonization compared with placebo	No serious adverse events were observed	(65)

*Dose categories were defined for descriptive comparison and do not represent formal clinical recommendations. Weekly regimens were converted to daily equivalents for classification purposes.

### 5.2 Comparison of topical versus systemic vitamin D therapies in AD

Both topical and systemic vitamin D therapies have been explored for the treatment of AD, each with its own advantages and limitations. Systemic vitamin D supplementation has shown promise in some studies (**Table 1**). A RCT in patients with severe AD found that vitamin D3 supplementation at 1600 IU/day, along with baseline therapy of topical 1% hydrocortisone cream, led to a significant increase in 25(OH)D levels at week 12. The mean EASI score was significantly lower in the treatment group compared to the placebo group, and the percent change in EASI score from baseline also decrease significantly in the treatment group, indicating that vitamin D supplementation could be an effective adjuvant treatment in severe AD (59). Topical vitamin D therapies, on the other hand, can directly target the skin. A study on an experimental model of AD in mice found that topical vitamin D3 cream (0.0003%) had a marked improvement in the skin of OVA-induced AD mice. It decreased the levels of IgE, IL-4, IL-13, IL-5 and epidermal thickness, while increasing the levels of filaggrin (67). However, the use of topical vitamin D in humans should be cautious, as in some cases, it may cause skin irritation or other adverse effects (68). Additionally, the penetration and systemic absorption of topical vitamin D need to be carefully considered. Overall, more research is needed to directly compare the efficacy, safety, and long-term effects of topical and systemic vitamin D therapies in AD patients.

### 5.3 Combination therapies involving vitamin D for AD management

Combination therapies that include vitamin D have been investigated to enhance the treatment of AD, taking advantage of the potential synergistic effects between different agents. One common approach is combining vitamin D supplementation with standard topical treatments. A study in children with AD found that when vitamin D was supplemented orally at 1000 IU/day along with emollient and patient education about AD and basic skin care, there was a significant improvement in the EASI score compared to placebo. This suggests that vitamin D can enhance the efficacy of basic skin care and emollient-based treatments (47). Another combination could be with other immunomodulatory agents. Since AD is characterized by an abnormal immune response, combining vitamin D with agents that target the immune system may have additive or synergistic effects. For example, as vitamin D can modulate the immune response by influencing T-cell subsets and cytokine production (69), combining it with drugs, such as topical corticosteroids or calcineurin inhibitors, that also regulate the immune system may lead to better disease control. However, the optimal combination, dosing, and treatment duration need to be further explored through well-designed clinical trials. Additionally, potential interactions between different agents in combination therapies should be carefully monitored to ensure safety and efficacy.

## 6 Controversies and future directions in vitamin D research for AD

### 6.1 Debates on optimal vitamin D dosage for AD patients

Determining the optimal vitamin D dosage for AD patients remains a subject of debate, with different studies suggesting varying results. Some studies have explored relatively high doses of vitamin D supplementation. A study in children with AD considered a dose of 2400 IU of vitamin D for those with severe AD (SCORAD index > 40) to achieve a comparable reduction in disease severity as in mild AD cases (70). However, other research has not found a clear-cut relationship between higher doses and better outcomes. A systematic review and meta-analysis of vitamin D supplementation in AD treatment found that the effectiveness of vitamin D supplementation may depend on factors such as geographic location and the age group of the patients (71). The lack of consensus on the optimal dosage may be due to several factors. Patient-specific factors, such as genetic polymorphisms in genes related to vitamin D metabolism and action, can influence the response to different doses. Additionally, the baseline vitamin D status of the patients, their sun exposure, and dietary habits can also play a role. Moreover, the heterogeneity of AD itself, in terms of disease severity, phenotype and associated comorbidities, may contribute to the variability in the response to vitamin D supplementation. Well-designed, large-scale, RCTs are needed to determine the optimal vitamin D dosage for different subgroups of AD patients.

### 6.2 Limitations of current clinical evidence on vitamin D in AD

Despite the growing interest in the role of vitamin D in AD, there are several limitations in the current clinical evidence. One major limitation is the heterogeneity of study designs. Different studies have used various inclusion and exclusion criteria, patient populations, dosing regimens, and outcome measures. For example, some studies focus on pediatric patients, while others include adults; some use high-dose vitamin D supplementation, while others use lower doses. This makes it difficult to compare and pool the results, leading to inconsistent findings. A systematic review of studies on the effects of vitamin D levels and supplementation on AD found that the included papers were heterogeneous in terms of location, season, and vitamin D supplementation regimen, which may have influenced the results (72).

Another limitation is the relatively small sample sizes in many studies. Small sample sizes may not have enough statistical power to detect subtle but clinically relevant differences, especially when it comes to rare phenotypes or milder forms of AD. For instance, a study on the association between vitamin D deficiency and acne, which is also an inflammatory skin disease, had a small number of patients in the supplementation study, limiting the generalizability of the results (73).

Furthermore, confounding factors are often not fully controlled. Factors such as sun exposure, diet, and other comorbidities can influence both vitamin D levels and AD severity. In a study on the relationship between serum vitamin D levels and AD, factors like sunlight exposure, vitamin D intake, and AD treatment were not adequately accounted for, potentially confounding the relationship between serum 25(OH)D and AD severity (74).

### 6.3 Emerging research on vitamin D in AD treatment

Emerging research on vitamin D in AD treatment is exploring new mechanisms and potential therapeutic approaches. One area of focus is the role of vitamin D in modulating the skin microbiome. As the skin microbiome is dysbiosis in AD, with an over-representation of *S. aureus*, understanding how vitamin D can interact with the skin microbiota may lead to new treatment strategies. Vitamin D can enhance the production of AMPs, which may help control the growth of pathogenic bacteria on the skin. Additionally, it may influence the composition of the skin microbiota by modulating the immune response in the skin, creating an environment less favorable for the over-growth of harmful bacteria (75). Another emerging area is the potential integration of vitamin D supplementation with targeted biologic therapies in AD. Biologics such as dupilumab inhibit downstream Th2 cytokine signaling (IL-4/IL-13), whereas vitamin D may modulate upstream epithelial and immune pathways. Notably, vitamin D has been shown to regulate DNA methylation and reduce thymic stromal lymphopoietin (TSLP) gene expression (76). As TSLP is a key initiator of Th2 inflammation and a therapeutic target in allergic diseases (77), this upstream modulation may provide mechanistic complementarity with biologic therapies. Although clinical evidence for combination strategies remains limited, this model offers a biologically plausible rationale for further investigation. Furthermore, research is also exploring the role of vitamin D in the early prevention of AD. Some studies suggest that vitamin D status during pregnancy or early infancy may influence the development of AD later in life. Understanding these early-life effects of vitamin D could potentially lead to preventive strategies, such as targeted vitamin D supplementation during pregnancy or infancy to reduce the risk of AD development.

### 6.4 Future perspectives on vitamin D and AD research

Future research on vitamin D and AD holds several promising directions. Firstly, more large-scale, well-designed RCTs are needed. These trials should have homogeneous patient populations, clear inclusion and exclusion criteria, and standardized dosing regimens. They should also measure multiple outcomes, including disease severity, quality of life, and biomarker changes, to comprehensively evaluate the role of vitamin D in AD. For example, an RCT could be designed to specifically investigate the long-term effects of different vitamin D dosages on AD severity, recurrence rates, and the development of comorbidities in a well-defined patient population. Secondly and importantly, emerging evidence linking VDR polymorphisms, such as *BsmI*, *ApaI*, and *TaqI*, to AD susceptibility and immune responsiveness highlights the need for genotype-directed therapeutic strategies. Genetic variation in VDR may influence receptor function, downstream signaling efficiency, and individual responsiveness to vitamin D supplementation. Future trials should therefore incorporate genetic stratification to determine whether specific VDR genotypes are associated with differential clinical responses to vitamin D. Such genotype-informed study designs could pave the way for precision-based vitamin D therapy in AD, where dosing and treatment duration are tailored according to genetic background. Thirdly, exploring the long-term safety of vitamin D supplementation and therapies is essential. As vitamin D is involved in many physiological processes, long-term use of high doses or inappropriate use may have potential side effects, such as hypercalcemia. Future studies should carefully monitor these potential adverse effects over extended periods to ensure the safe use of vitamin D-based therapies in AD patients. Additionally, research on the interactions between vitamin D and other medications commonly used in AD treatment is needed to avoid potential drug-drug interactions.

## 7 Conclusion

Vitamin D has emerged as a multifaceted regulator of AD, influencing epidermal differentiation, skin barrier integrity, immune cell function, and microbial homeostasis. Through its action on the VDR and associated signaling pathways, vitamin D modulates the expression of AMPs, pro- and anti-inflammatory cytokines, and genes involved in skin homeostasis. Epidemiological and clinical studies support an association between vitamin D deficiency and increased AD severity, although results from supplementation trials remain inconsistent. Factors such as genetic variability, baseline vitamin D status, age, and geographic region may influence therapeutic outcomes. Future research should prioritize well-designed, large-scale RCTs to clarify optimal dosing strategies and identify subgroups that may benefit most from vitamin D-based therapies. Moreover, the integration of omics-based biomarkers and microbiome analyses will be key to developing personalized treatment strategies. Overall, targeting the vitamin D signaling axis represents a promising adjunctive approach in the management of AD.
